# Associations between hemispheric asymmetry and schizophrenia-related risk genes in people with schizophrenia and people at a genetic high risk of schizophrenia

**DOI:** 10.1192/bjp.2021.47

**Published:** 2021-07

**Authors:** Yue Zhu, Shuai Wang, Xiaohong Gong, Elliot K. Edmiston, Suyu Zhong, Chao Li, Pengfei Zhao, Shengnan Wei, Xiaowei Jiang, Yue Qin, Jujiao Kang, Yi Wang, Qikun Sun, Gaolang Gong, Fei Wang, Yanqing Tang

**Affiliations:** 1 Department of Psychiatry, The First Affiliated Hospital of China Medical University, PR China; and Brain Function Research Section, The First Affiliated Hospital of China Medical University, PR China; 2 Department of Psychiatry, The First Affiliated Hospital of China Medical University, PR China; Brain Function Research Section, The First Affiliated Hospital of China Medical University, PR China; and Department of Psychology, Weifang Medical University, PR China; 3 State Key Laboratory of Genetic Engineering and Human Phenome Institute, School of Life Sciences, Fudan University, PR China; 4 School of Medicine, University of Pittsburgh, USA; 5 State Key Laboratory of Cognitive Neuroscience and Learning & IDG/McGovern Institute for Brain Research, Beijing Normal University, PR China; 6 Brain Function Research Section, The First Affiliated Hospital of China Medical University, PR China; and Department of Radiology, The First Affiliated Hospital of China Medical University, PR China; 7 Department of Radiation Oncology, The First Affiliated Hospital of China Medical University, PR China; 8 Department of Psychiatry, The First Affiliated Hospital of China Medical University, PR China; Brain Function Research Section, The First Affiliated Hospital of China Medical University, PR China; Department of Radiology, The First Affiliated Hospital of China Medical University, PR China; and Department of Psychiatry, Yale School of Medicine, USA; 9 Department of Psychiatry, The First Affiliated Hospital of China Medical University, PR China; Brain Function Research Section, The First Affiliated Hospital of China Medical University, PR China; and Department of Geriatrics, The First Affiliated Hospital of China Medical University, PR China

**Keywords:** Structural network, brain asymmetry, polygenic risk score, genetic susceptibility, schizophrenia

## Abstract

**Background:**

Schizophrenia is considered a polygenic disorder. People with schizophrenia and those with genetic high risk of schizophrenia (GHR) have presented with similar neurodevelopmental deficits in hemispheric asymmetry. The potential associations between neurodevelopmental abnormalities and schizophrenia-related risk genes in both schizophrenia and those with GHR remains unclear.

**Aims:**

To investigate the shared and specific alternations to the structural network in people with schizophrenia and those with GHR. And to identify an association between vulnerable structural network alternation and schizophrenia-related risk genes.

**Method:**

A total of 97 participants with schizophrenia, 79 participants with GHR and 192 healthy controls, underwent diffusion tensor imaging (DTI) scans at a single site. We used graph theory to characterise hemispheric and whole-brain structural network topological metrics. For 26 people in the schizophrenia group and 48 in the GHR group with DTI scans we also calculated their schizophrenia-related polygenic risk scores (SZ-PRSs). The correlations between alterations to the structural network and SZ-PRSs were calculated. Based on the identified genetic–neural association, bioinformatics enrichment was explored.

**Results:**

There were significant hemispheric asymmetric deficits of nodal efficiency, global and local efficiency in the schizophrenia and GHR groups. Hemispheric asymmetric deficit of local efficiency was significantly positively correlated with SZ-PRSs in the schizophrenia and GHR groups. Bioinformatics enrichment analysis showed that these risk genes may be linked to signal transduction, neural development and neuron structure. The schizophrenia group showed a significant decrease in the whole-brain structural network.

**Conclusions:**

The shared asymmetric deficits in people with schizophrenia and those with GHR, and the association between anomalous asymmetry and SZ-PRSs suggested a vulnerability imaging marker regulated by schizophrenia-related risk genes. Our findings provide new insights into asymmetry regulated by risk genes and provides a better understanding of the genetic–neural pathological underpinnings of schizophrenia.

## Background

Schizophrenia is a highly heritable disorder with neurodevelopmental deficits – characterised by a failure to integrate neural processes as a result of an abnormal brain network.^1–3^ These brain network deficits can occur years before the illness appears through disruptions of normal neuro-maturational processes. Furthermore, family studies reported a tenfold increased risk of developing schizophrenia in unaffected relatives of people with schizophrenia,^4^ and polygenic risk score (PRS) analysis was estimated to explain 7% of the variance in liability.^5^ Identifying these potential imaging features could help explain the multilevel genetic–neural underpinnings of the development of schizophrenia.

## Hemispheric asymmetry and schizophrenia

Neurodevelopmental researchers have suggested that brain asymmetry is a core metric of both neurodevelopment in healthy individuals^6,7^ and in numerous developmental disorders.^8,9^ Previous post-mortem and neuroimaging studies in schizophrenia have repeatedly shown the condition to be associated with an anomalous pattern of hemispheric asymmetry.^10–13^ Crow and colleagues undertook a series of studies^14,15^ and proposed an influential theory: schizophrenia, which stems from the failure of normal hemispheric asymmetry in the temporal lobe region can be explained by genes.^15^ Furthermore, Crow et al indicated that hemispheric asymmetry in schizophrenia was unrelated to episode progression, and the anatomical asymmetry occurred during development.^10^ These neurodevelopmental alterations in hemispheric asymmetry were found both in brain structure and function, such as white matter architecture, cortical thickness and functional activation.

## White matter

White matter plays a pivotal role in modulating communication and the functional integrity of the brain. A brain network study found that reduced communication capacity and altered functional brain dynamics in schizophrenia may be caused by a selective disruption of brain connectivity among central hub regions of the brain.^16^ Anomalous white matter integrity has been associated with neurodevelopmental abnormalities of myelination, axonal growth and synaptic plasticity in schizophrenia.^17,18^ Pathological turbulences of the brain are rarely focused on a single brain region, as deficits often spread via axonal pathways to affect other regions.

Using diffusion tensor imaging (DTI), we can virtually reconstruct white matter tracts and model the human brain as a complex network/graph. Graph theoretical analysis can provide a powerful new way to evaluate the topological organisation of the constructed human brain white matter network. Multiple topological metrics can be used to assess white matter network connectivity by graph theoretical analysis,^19,20^ which is a relatively novel technique to understand the neuropathology in neural disease. Neuroimaging evidence indicates a global efficiency decrease in the topological metric of brain anatomical networks in schizophrenia.^21^ Although some studies have focused on abnormal topological metrics of the whole-brain network, few studies have investigated alterations to topological metrics of hemispheric asymmetry and the whole-brain network within the context of the same study in schizophrenia.

## Benefits of studying populations at genetic high risk for developing schizophrenia

Abnormalities of hemispheric asymmetry and the whole-brain network have also been found in individuals without psychosis but with genetic high risk for developing schizophrenia (GHR).^20,22^ Thus, GHR populations can be used to identify liabilities expressed across a range of phenotypes, presumably reflecting vulnerability. Studies of morphology and the white matter network in neonates at genetic risk of schizophrenia indicated that this risk would induce lower efficiency both in the white matter network and grey matter structural associations.^23^ Furthermore, recent studies found that people with schizophrenia and their unaffected siblings showed disrupted asymmetry of inter- and intrahemispheric functional connectivity.^24^ A similar pattern was also found in prefrontal, occipitoparietal cortical regions compared with healthy controls,^25,26^ particularly, left-sided language dysfunctional asymmetry was considered to be the result of familial heritable outcomes.^27,28^ Evidence shows that people with schizophrenia and their unaffected monozygotic co-twins present with decreased language asymmetry; however, the asymmetry was not associated with the severity of psychosis, which suggested that asymmetry was a result of genetic risk, rather than a state-related trait.^29^ However, few studies have focused on evaluating white matter structural network topological metrics at the hemispheric level in people with schizophrenia and their unaffected relatives.

## Value of using PRSs

Although family association studies have confirmed genetics contributes significantly to schizophrenia risk, these studies have resulted in few replicated findings. It is difficult to find research on the latent genetic architecture of schizophrenia using individual single nucleotide polymorphisms (SNPs). This is because the disease is highly polygenic and has many common genetic variants facilitating the disease.^30^ Genome-wide association studies (GWASs) could identify millions of SNPs across the entire genome associated with psychiatric disorders. PRS analysis calculates a single score to predict disease risk, via combining risk alleles at thousands of genetic loci, and it provides a robust technique to investigate an individual's genetic risk for polygenic traits at a population level. These cumulative risk scores are based on the identification of genetic variants through GWASs.^31^ A family study found a positive correlation between the PRS of schizophrenia (SZ-PRS) and bilateral frontal gyrification, which implicated that SZ-PRSs had a negative effect on early neurodevelopment and enhanced the risk of developing the disorder.^32^ Furthermore, schizophrenia-related PRS was associated with early endogenous phenotypic alterations of neurofunction.^33–36^ Terwisscha van Scheltinga and colleagues described how higher PRS was related to smaller white matter volume, and suggested genetic schizophrenia-associated variants modulated white matter development.^37^ Therefore, PRS analysis makes it possible to test whether individuals with high familial risk of schizophrenia carry an increased burden of neurodevelopmental deficits. Bioinformatics enrichment analysis has been regarded as a promising tool that contributes to the gene functional analysis of large gene lists for various high-throughput biological studies. Fromer et al have indicated that polygenes played a role in synaptic transmissions that were enriched for schizophrenia genetic associations.^38^

## Aims

To the best of our knowledge, this is the first study to investigate the shared and specific alterations in topological metrics of hemispheric asymmetry and the whole-brain structural network in people with schizophrenia and those with GHR. We combined genetic imaging data (genetic variable × white matter structural network in hemispheric asymmetry and whole brain) to explore a vulnerability imaging marker regulated by schizophrenia-related risk genes. The primary aim of this study was to identify an association between shared white matter structural network alternation and schizophrenia-related risk genes in people with schizophrenia and those with GHR, and investigate the functions of these risk genes through bioinformatics enrichment analyses. Secondly, our aim was to reveal a core deficit in the white matter structural network related to pathology in schizophrenia.

## Method

### Participants

A total of 368 individuals participated in this study, including 97 people with schizophrenia, 79 with GHR and 192 healthy controls, aged 18–54 years. Detailed inclusion and exclusion criteria are described in the Supplementary Material available at https://doi.org/10.1192/bjp.2021.47. Symptom severity was measured using the 17-item version of the Hamilton Rating Scale for Depression (HRSD-17),^39^ the Hamilton Rating Scale for Anxiety (HRSA)^40^ and the Brief Psychiatric Rating Scale (BPRS).^41^ All participants gave written informed consent. This research was approved by the Medical Research Ethics Committee of the China Medical University and in accordance with the Declaration of Helsinki.

### Magnetic resonance imaging (MRI) data

#### MRI acquisition

All MRI scans were performed using a 3.0 T GE Sigma system (General Electric, Milwaukee, USA) with a standard eight-channel head coil at the First Affiliated Hospital of China Medical University, Shenyang, China. The parameters of *T*_1_ images and DTI are described in the Supplementary Material. Two neuro-radiologists with more than 3 years of experience interpreting neuroradiology images checked image quality.

#### Data preprocessing and network construction

The DTI data-set was preprocessed using PANDA.^42^ Briefly, data preprocessing included (a) brain extraction (b) correction for eddy-current distortion and simple head motion, (c) correction for b-matrix, and (d) computation for diffusion tensor and fractional anisotropy. The construction of the white matter network was implemented by PANDA. The procedures used for the white matter network construction are described in the Supplementary Material.

#### Network analysis

Graph theory was used to characterise the topological metrics of the white matter structural networks derived above. In the current study, both nodal metrics and global network metrics were computed. We characterised a single nodal metric by computing the nodal degree (D_nodal_) and nodal efficiency (E_nodal_). The global metrics of the network were computed for the global efficiency (E_glob_) and local efficiency (E_loc_). GRETNA (https://www.nitrc.org/projects/gretna/) was used to calculate network metrics.^43^ Brief descriptions and formulas are provided in Supplementary Table 1.

#### Asymmetry index analysis

White matter structural network asymmetry of topological metrics (D_nodal_, E_nodal_, E_glob_ and E_loc_) was estimated using the asymmetry index (AI): AI(X) = 100 × [X(L) – X(R)]/[X(L) + X(R)], where X(L) and X(R), respectively, represent the network metrics of the left and right hemispheres. AI provides the differences between the left and right hemispheres, by incorporating the relative network metrics over both hemispheres in one value.

### Genetic data

#### Genotyping and imputation

Whole blood samples were withdrawn into EDTA (ethylenediaminetetraacetic acid) anticoagulant tubes, with samples taken between 10.00 h and 15.00 h and stored at −80°C until it was assayed. Genomic DNA was extracted from whole blood using standard protocols. Illumina Global Screening Array-24 v1.0 BeadChip was used to screen genome-wide variants for 74 participants (26 in the schizophrenia group and 48 in the GHR group). Detailed demographic and clinical data are provided in Supplementary Table 2 for the 74 participants included in the genetic analysis. This array provides data for 642 824 fixed genetic variants, addition to 53 411 customised variants. Detailed exclusion criteria relating to data and genotype imputation are described in the Supplementary Material.

#### Calculation of PRSs

The latest international GWAS results published by the Psychiatric Genomics Consortium were used as discovery samples, and our imputed genotyping data were used as a target sample. In the paper by the Bipolar Disorder and Schizophrenia Working Group of the Psychiatric Genomics Consortium,^44^ the specific genetic factors contributing to schizophrenia were analysed in 33 426 people with schizophrenia and 32 541 controls. A total of 843 107 ambiguous variants were excluded. PRSs were generated using PRSice software (www.PRSice.info). *P*-value-informed clumping was performed with a cut-off of *r*^2^ = 0.1 in a 250 kb window. Twelve PRSs at different *P*-value thresholds (0.0001, 0.001, 0.01, 0.02, 0.03, 0.04, 0.05, 0.1, 0.2, 0.3, 0.4 and 0.5) were derived for each study participant. The number of variants for 12 PRSs were 915, 2523, 6376, 8055, 9185, 10 102, 10 846, 13 638, 17 339, 20 086, 22 299 and 24 268, respectively.

#### Bioinformatics enrichment analyses

All the SNPs in SZ-PRSs under a certain *P*-value threshold were extracted and transformed into the corresponding genes where they were located based on the dbSNP database. A gene list was obtained and uploaded to the online tool DAVID Bioinformatics Resources v6.8 (https://david.ncifcrf.gov/)^42^,^43^ for the Gene Ontology and the Kyoto Encyclopedia of Genes and Genomes (KEGG) pathway analyses. The functions of genes were annotated with three Gene Ontology terms: biological process, cellular component and molecular function. Multiple testing corrections were performed with the Bonferroni method (significance level at 0.01).

### Statistical analysis

ANOVAs (analyses of variance) or chi-square tests were used to examine participants’ demographic characteristics (age and gender) and clinical characteristics (duration of illness, first episode and medication status). ANCOVA (analyses of covariance) was implemented to evaluate differences between white matter network topological metrics for the asymmetry index and the entire brain among the three groups, with gender and age as covariates. Least significant differences *post hoc* analyses were performed to detect significant group effects in the ANCOVA. Bonferroni correction was applied for multiple comparisons (90 tests), and significance was set to a corrected *P* < 0.05. Partial correlation analyses, with age and gender as covariates, were performed to investigate the relationships between asymmetry index metrics and entire brain network metrics with SZ-PRSs in the schizophrenia and GHR groups. Significance was set at *P* < 0.05 (two-tailed) for all tests. All analyses were performed using SPSS 22.0.

## Results

### Demographics and clinical scales

No significant between-group differences were found in age and gender. The effect of diagnosis on HRSD, HRSA and BPRS scores was significant (see Table 1), with significantly higher HRSD, HRSA and BPRS scores in the schizophrenia group compared with the GHR and healthy control groups, and higher HRSD scores in the GHR group compared with the healthy control group. There was no significant difference between the GHR and healthy control groups in HRSA and BPRS.

**Table 1:** Demographic and clinical characteristics of the schizophrenia, genetic high risk of schizophrenia (GHR) and healthy control groups

	Schizophrenia group	GHR group	Healthy control group	*F*/χ^2^	*P*
*n*	97	79	192	–	–
Demographic characteristics					
Age, years: mean (s.d.)	29.81 (9.40)	29.52 (7.72)	31.22 (9.85)	1.281	0.279
Female, *n* (%)	55(56.7)	34 (43.0)	107 (55.7)	4.248	0.120
Clinical characteristics					
Duration of illness, months: mean (s.d.)	36.69 (48.93)	—	—	–	–
First episode, yes: *n* (%)	51(53.7)	—	—	–	–
Medication, yes: *n* (%)	69 (71.1)	—	—	–	–
Hamilton Rating Scale for Depression, mean (s.d.)	7.23 (6.35)	2.53 (3.80)	1.19 (2.26)	63.568	<0.001
Hamilton Rating Scale for Anxiety, mean (s.d.)	6.03 (7.15)	2.03 (4.37)	1.00 (2.52)	32.693	<0.001
Brief Psychiatric Rating Scale, mean (s.d.)	31.13 (11.39)	18.92 (2.22)	18.63 (1.93)	118.038	<0.001

### Asymmetry index among the schizophrenia, GHR and healthy control groups

**Fig. 1:**
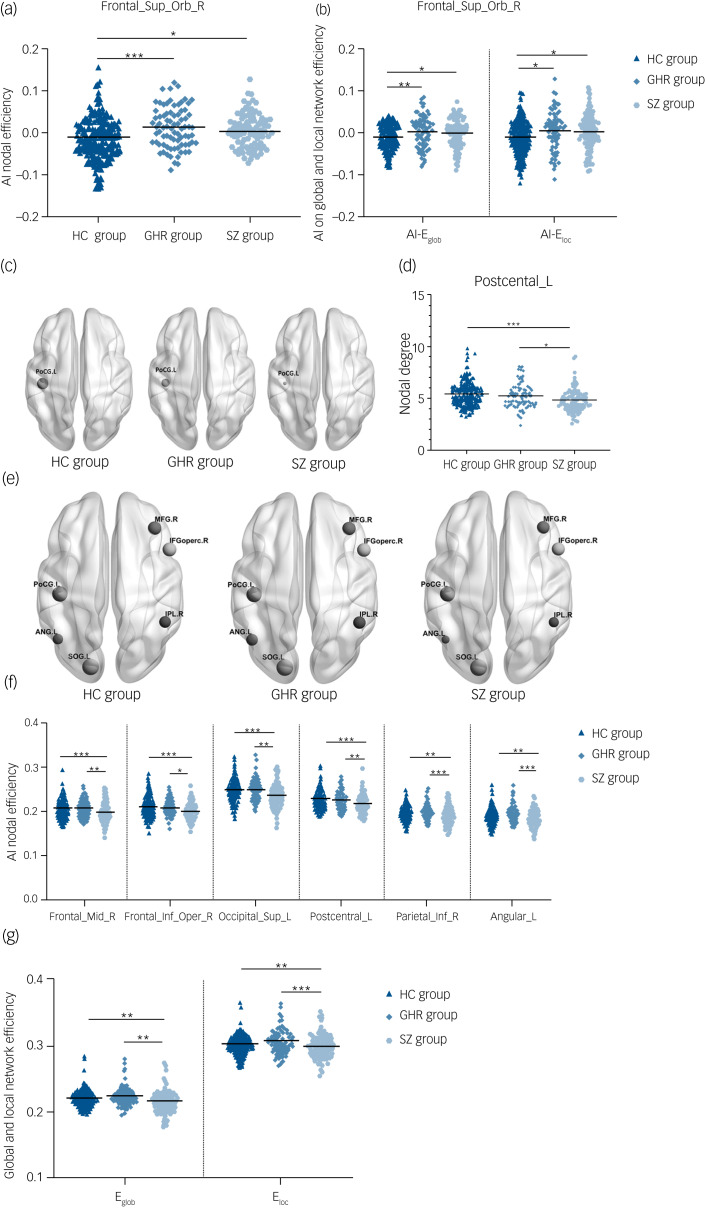
(a) AI-E_nodal_ among the schizophrenia, GHR and healthy control groups. (b) AI-E_glob_ and AI-E_loc_ among the schizophrenia, GHR and healthy control groups. (c) Three-dimensional representations of the D_nodal_ in the entire brain among the schizophrenia, GHR and healthy control groups. (d) D_nodal_ in the entire brain among the schizophrenia, GHR and healthy control groups. (e) Three-dimensional representations of the E_nodal_ in the entire brain among schizophrenia, GHR and healthy control groups. (f) E_nodal_ in the entire brain among schizophrenia, GHR and healthy control groups. (g) E_glob_ and E_loc_ in the entire brain among schizophrenia, GHR and healthy control groups. AI, asymmetry index; ANG.L and Angular_L, left postcentral gyrus; D_nodal_, nodal degree; E_glob_, global efficiency; E_loc_, local efficiency; E_nodal_, nodal efficiency; Frontal_Sup_Orb_R, right superior frontal gyrus, orbital part; IFGoperc.R and Frontal_Inf_Oper_R, right inferior frontal gyrus, opercular part; IPL.R and Parietal_Inf_R, right inferior parietal angular gyrus; HC, healthy control; GHR, genetic high risk of schizophrenia; PoCG.L and Postcentral_L, left postcentral gyrus; SOG.L and Occipital_Sup_L, left superior occipital gyrus; MFG.R and Frontal_Mid_R, right middle frontal gyrus; SZ, schizophrenia. ***P < 0.001; **P < 0.01; *P < 0.05.

Significant group effects were observed in AI-E_nodal_ of the right orbital superior frontal gyrus in the three groups. *Post hoc* analysis revealed significant increases in the schizophrenia and GHR groups compared with the healthy control group. There was no statistical difference between schizophrenia and GHR (details in Fig. 1(a) and Supplementary Table 3). ANCOVA showed significant between-group effects in AI-E_glob_ and E_loc_ among the schizophrenia, GHR and healthy control groups. Comparisons of the healthy control, schizophrenia and GHR groups increased in AI-E_glob_ and E_loc_ but did not differ from each other (details in Fig. 1(b) and Supplementary Table 3). No significant group effect was found in AI-D_nodal_ after Bonferroni correction among the schizophrenia, GHR and healthy control groups.

### D_nodal_ and E_nodal_ of whole brain among the schizophrenia, GHR and healthy control groups

A significant difference was observed in D_nodal_ left postcentral gyrus, and *post hoc* analyses revealed that compared with the healthy control and GHR groups, the schizophrenia group showed a decrease in D_nodal_. Significant group differences in the E_nodal_ of the right middle frontal gyrus, right opercular inferior frontal gyrus, left superior occipital gyrus, left postcentral gyrus, right inferior parietal angular gyrus and left angular gyrus were observed. *Post hoc* analyses revealed that compared with the healthy control and GHR groups, the schizophrenia group showed a decrease in E_nodal_. But there was no significant difference in D_nodal_ and E_nodal_ between the GHR and healthy control groups (details in Fig. 1(d), 1(f) and Supplementary Table 3).

### E_glob_ and E_loc_ of whole brain among the schizophrenia, GHR and healthy control groups

Significant differences were found in E_glob_ and E_loc_ among the three groups. Compared with the healthy control and GHR groups, the schizophrenia group showed a decrease in E_glob_ and E_loc_, but there was no significant difference between the GHR and healthy control groups (Fig. 1(g) and Supplementary Table 3).

### Correlation between SZ-PRS and AI-E_loc_ in the schizophrenia and GHR groups

We excluded participants who failed to evaluate SZ-PRS, and demographic and clinical data for participants in the correlation analysis are provided in Supplementary Table 2. According to the aims of our study, we performed partial correlation analyses to investigate the relationships between asymmetry index metrics and entire brain network metrics using SZ-PRS rather than using a gene-based association test to find specific risk genes influencing development of hemispheric asymmetry. AI-E_loc_ was significantly positively correlated with SZ-PRS at *P*-value thresholds of 0.0001, 0.001, 0.01, 0.02, 0.03, 0.04, 0.05, 0.1, 0.2, 0.3, 0.4 and 0.5, and after Bonferroni correction there was significant correlation at *P*-value thresholds of 0.03, 0.04, 0.05, and 0.1 (Table 2). There was no significant correlation between other white matter network or asymmetry index metrics with SZ-PRSs.

**Table 2:** Association of schizophrenia-related polygenic risk score (SZ-PRS) with asymmetry index (AI)- local efficiency (E_loc_) in the schizophrenia and genetic high risk of schizophrenia (GHR) groups

PT	0.0001	0.001	0.01	0.02	0.03	0.04	0.05	0.1	0.2	0.3	0.4	0.5
AI-E_loc_												
*r*	0.293	0.317	0.267	0.318	0.369	0.39	0.391	0.34	0.271	0.223	0.236	0.234
*P*	1.25E−02	6.67E−03*	2.36E−02**	6.45E−03*	1.45E−03**	**7.04E**−04**	**6.87E**−04**	**3.49E**−03**	2.11E−02**	5.98E−02*	4.57E−02	4.75E−02*

PT, *P*-value thresholds.

Bold indicates significance at *P* < 0.05, after Bonferroni correction.

***P < 0.001; **P < 0.01; *P < 0.05.

### Gene Ontology and KEGG pathway enrichment analyses for genes of SZ-PRSs

**Fig. 2:**
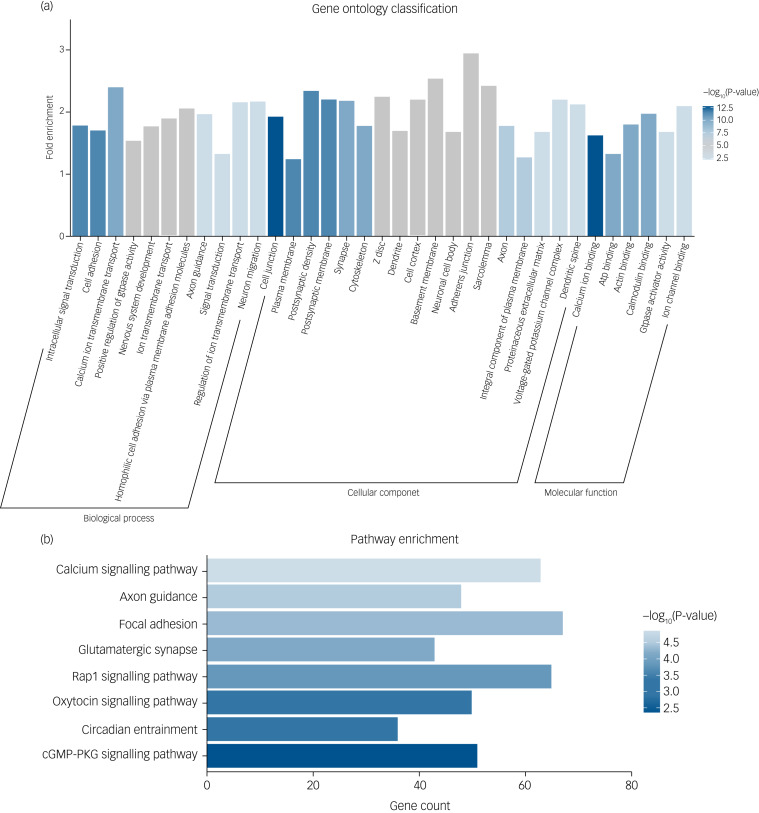
(a) Significant Gene Ontology enrichment analysis for risk genes of schizophrenia-related polygenic risk scores (SZ-PRSs). (b) Significant Kyoto Encyclopedia of Genes and Genomes (KEGG) pathway enrichment analysis for risk genes of SZ-PRSs. cGMP-PKG, cyclic guanosine monophosphate-protein kinase G.

To explore the biological mechanism of SZ-PRS genes involved in schizophrenia, we conducted bioinformatics enrichment analyses for genes of SZ-PRSs at *P-*value thresholds of 0.05 (PT_0.05), which has the smallest *P*-value in the association analysis with *AI-*E*_loc_*. In total, 10 729 SNPs and 4070 genes were extracted and identified in SZ-PRSs at PT_0.05. Thirty-five Gene Ontology terms were detected for SZ-PRS genes (Fig. 2a).

For biological process, terms were enriched in signal transduction (intracellular signal transduction, calcium ion transmembrane transport, ion transmembrane transport and positive regulation of GTPase activity) as well as neural development (cell adhesion, nervous system development and axon guidance).

With regard to cellular components, neuron structure was among the significant aspects, containing several terms (cell junction, plasma membrane, postsynaptic density, postsynaptic membrane and cytoskeleton).

Calcium ion binding was the most significant term in molecular function. Additionally, we obtained eight significantly enriched pathways (Fig. 2b) based on the KEGG database, including calcium signalling pathway, axon guidance, focal adhesion, glutamatergic synapse, Rap1 signalling pathway and oxytocin signalling pathway.

## Discussion

### Main findings

To our knowledge, this is the first combined genetic imaging study (genetic variable × white matter network in hemispheric asymmetry and the entire brain) to investigate shared and specific alterations in hemispheric asymmetry and the whole-brain structural network among people with schizophrenia, those with GHR and healthy controls, to estimate the possible effects of genes on the white matter network, and to explore SZ-PRS bioinformatics enrichment. There were shared alterations in network topological asymmetry (right orbital superior frontal gyrus of E_nodal_, E_glob_ and E_loc_) in the schizophrenia and GHR groups. The implication of this is that genetic susceptibility to schizophrenia potentially regulated abnormalities in the cerebral hemispheres. Furthermore, we found E_loc_ of hemispheric asymmetry was associated with SZ-PRSs in both the schizophrenia and GHR groups, and bioinformatics enrichment analyses revealed that genes driving the SZ-PRS interaction were involved in signal transduction, neural development, neuron structure and calcium signalling pathways. Thus, we were able to link our imaging findings to potential pathways involved in the molecular pathophysiology of schizophrenia.

In the schizophrenia group, we observed decreased D_nodal_ (left postcentral gyrus), E_nodal_ (right middle frontal gyrus, right opercular inferior frontal gyrus, left superior occipital gyrus, left postcentral gyrus, right inferior parietal angular gyrus and left angular gyrus), E_glob_ and E_loc_ of the entire brain, compared with the GHR and healthy control groups.

### Interpretation of our findings and comparison with findings from other studies

The human brain is structurally and functionally asymmetrical – the left cerebral hemisphere is typically associated with language ability, and the right hemisphere is typically associated with non-verbal functions.^7,47,48^ Although the cerebral hemispheres are similar in weight and volume, there is a difference in brain tissue distribution. The right hemisphere protrudes anteriorly beyond the left, and the left hemisphere extends posteriorly beyond the right.^47,49^ In addition, the right hemisphere is significantly more efficient and interconnected than the left, whereas the left hemisphere has more central/indispensable regions for whole-brain structural network function.^50^

These results are in line with brain functional principles: the left hemisphere may demand specialised networks for processes such as language and motor actions, whereas the right hemisphere is more efficient and interconnected for more general processes such as integrating information. Patients with schizophrenia do not show these patterns of right-more-than-left efficient global integration that are observable in healthy control participants. Consistent with this result, our study found that patients with schizophrenia and those with GHR have higher asymmetry index scores in E_glob_ and E_loc_ (AI = L-R/L + R), implying that in both schizophrenia and those with GHR there is abnormal lateralisation at the ‘whole-hemisphere’ level. Furthermore, those with schizophrenia demonstrated lower global and local efficiency in the whole brain and higher asymmetry index scores in the ‘whole hemisphere’. These findings suggest that there may be right hemisphere impairments of efficient connection in schizophrenia.

We also found local network efficiency of asymmetry to be positively correlated with SZ-PRSs. This finding suggested that schizophrenia-related risk genes may influence aberrant alterations in hemispheric asymmetry. This finding is also in line with a previous study, in which higher SZ-PRSs were associated with a steeper decline in the white matter network in older age.^51^ Although enormous studies have illustrated functional and structural network differences in the two hemispheres and genetic risk factors have contributed to the development of abnormal lateralisation, post-mortem studies have failed to find hemisphere discrepancies of gene expression in cerebral cortex.^52,53^ Due to the lack of availability of post-mortem tissue samples, these studies have small sample sizes (four mid-fetal brains and two adult brains, respectively). There may still be significant lateralised expression differences, particularly because it is likely that multiple genes interact to influence neuronal and circuit properties. Overall, our PRS findings may help to identify intermediate brain phenotypes that are fundamental or common in the development of schizophrenia neuropathology.

Interestingly, we conducted bioinformatics enrichment analyses to identify the functions of schizophrenia-related polygenic risk that influenced aberrant alterations in hemispheric asymmetry. We found genes driving the SZ-PRS interaction were involved in functions such as signal transduction, neural development, neuron structure and calcium signalling pathways. One study has found that brain asymmetry was regulated by genes, and this asymmetrical genes expression was involved in signal transduction, synaptic plasticity and axonal guidance.^54^ Lateralization of gene expression in language cortex has identified genes that can fine-tune electrophysiology and neurotransmission of cortical circuits through synaptic transmission, signal transduction, glutamate receptor activity, nervous system development, system development, transmission of nerve impulse and multicellular organismal development.^55^ Asymmetry of olfactory neurons in *Caenorhabditis elegans* (nematodes) was established by communicating via gap junctions, calcium signalling and tight junctions.^56^ Our findings contribute to the increasing evidence that multiple risk genes in schizophrenia help to explain anomalous brain asymmetry.

We also found a consistent decline in nodal degree and nodal efficiency of the left postcentral gyrus in the patients with schizophrenia. The major function of the postcentral gyrus is primary somatosensory processing, which includes somatotopic information as well as receipt of peripheral tactile and kinaesthetic sensation.^57^ Furthermore, the schizophrenia group showed lower E_glob_ and E_loc_ consistent with previous studies^3,16,21,58^ that have demonstrated disrupted connectivity in global and local white matter networks in schizophrenia. Additionally, there were no differences in the white matter structural network of the whole brain between the GHR and healthy control groups in this study. The specificity of the whole-brain structural network deficits in the schizophrenia group indicates that these deficits relate to the disorder.

### Limitations

There were several limitations in this study. First, most of the patients with schizophrenia were taking psychotropic medications at the time of study participation. Second, a moderate sample size was used for association analyses between SZ-PRSs and abnormal asymmetric changes (total 74 participants including participants with schizophrenia and those with GHR) in this study. Further study is needed, in a larger, unmedicated sample to confirm our results. Additionally, environmental risk factors could also influence the development of abnormalities in hemispheric asymmetry and the whole-brain structural network in schizophrenia. Therefore, future studies are needed to evaluate how specific genes and their interactions with environmental risks may contribute to the alterations in asymmetry found in schizophrenia.

### Implications

The shared deficits of hemispheric asymmetry in patients with schizophrenia and those with GHR suggested that anomalous asymmetry may be potential susceptibility markers of the disease. The significant association between altered hemispheric asymmetry and schizophrenia-related risk genes indicate a vulnerability imaging marker regulated by schizophrenia-related risk genes. These risk genes are also involved in signal transduction, neural development, neuron structure and calcium signalling pathways. These specific alterations to the white matter structural network of whole brain in people with schizophrenia largely relate to the neuropathologic features of the disorder. Our findings provide new insights into asymmetry regulated by risk genes and provide a better understanding of the genetic–neural pathological underpinnings of schizophrenia.

## Data Availability

The data that support the findings of this study are available from the corresponding author (Y.T.), upon reasonable request.

## Supplementary material

For supplementary material accompanying this paper visit http://dx.doi.org/10.1192/bjp.2021.47. click here to view supplementary material
