# Supplementary material for: Associations between hemispheric asymmetry and schizophrenia-related risk genes in people with schizophrenia and people at a genetic high risk of schizophrenia
Source: Br J Psychiatry. 2021 Jul;219(1):392–400. doi: 10.1192/bjp.2021.47 (PMC8529637; doi:10.1192/bjp.2021.47)
Supplement: Supplementary file 1 [file S0007125021000477sup001.docx]

**Supplementary Material**

Additional methods

*Participants*

In- and out- patients with SZ were recruited from the Department of Psychiatry at the First Affiliated Hospital of China Medical University and Shenyang Mental Health Center. Participants with GHR were all first-degree relatives of patients presenting with SZ at the Department of Psychiatry at the First Affiliated Hospital of China Medical University and Shenyang Mental Health Center. HCs were recruited from the local community using advertisements.

SZ participants met Diagnostic and Statistical Manual of Mental Disorders-IV-Text Revision (DSM-IV-TR) diagnostic criteria for SZ and the diagnoses were confirmed by two trained psychiatrists using the Structured Clinical Interview for DSM-IV Axis I disorders (SCID-I). GHR participants were all first-degree relatives of individuals with SZ who did not meet criteria for any DSM-IV Axis I disorder. HCs were individuals who did not have a current or previous history of Axis I disorders, and did not have any first-degree relatives with a history of Axis I disorders. Exclusion criteria for all participants included (1) substance/alcohol abuse or dependence or a concomitant major medical disorder, (2) significant pathological change found via T1- and T2-weighted MRI, (3) history of head trauma with loss of consciousness for ≥5 minutes or any neurological disorder, (4) any infectious diseases such as HIV/AIDS or severe acute respiratory syndrome, (5) any MRI contraindications, (6) suboptimal imaging data quality. The recruitment timeframe was from September 2009 to July 2018.

*MRI Acquisition*

T1 images were acquired using a three-dimensional fast spoiled gradient-echo sequence with the following parameters: TR/TE = 7.1/3.2 ms, image matrix = 240 × 240, field of view (FOV) = 240 × 240 mm2, 176 contiguous slices of 1 mm without gap, and voxel size = 1.0 × 1.0 × 1.0 mm3. Diffusion tensor imaging (DTI) was acquired using a single-short spin-echo planar imaging sequence with the following parameters: TR/TE = 17,000/85.4 ms, image matrix = 120 × 120, FOV = 240 × 240 mm2, 65 contiguous slices of 2 mm without gap, 25 noncollinear directions (b = 1000 s/mm2), together with an axial acquisition without diffusion weighting (b = 0), voxel size = 2.0 × 2.0 × 2.0 mm3.

*Data Preprocessing and* *Network Construction*

DTI data were processed using Pipeline for Analyzing braiN Diffusion imAges (PANDA) (http://www.nitrc.org/projects/panda), a fully automated program for processing brain diffusion images. We used default program parameters to process DTI images. Steps were as follows: 1) converting DICOM files into NIfTI images, 2) brain extraction, 3) correction for eddy-current distortion and head motion, 4) correction for b-matrix, 5) computation for diffusion tensor metrics. Next, diagonalization was performed to yield 3 pairs of eigenvalues and eigenvectors. Based on the 3 eigenvalues, fractional anisotropy (FA) was computed on a voxel-by-voxel basis. Then, PANDA nonlinearly registered individual FA images of native space to the FMRIB58_FA template in MNI (Montreal Neurological Institute) space with 2 mm3 voxels. The normalized FA is overlaid with image edges that were derived from the FA template. These pictures can be carefully viewed to check the quality of normalization. FA images were then smoothed with a 6mm Gaussian kernel. FA were chosen to examine because it could reflect the overall integrity of white matter, and a reduction in FA can reflect a decrease in myelination.

The construction of WM network was implemented by PANDA. The procedure consisted of two basic steps: the definitions of nodes and edges.

Network Node Definition The automated anatomical labeling atlas (AAL atlas) was used to parcellate the brain’s cortical and subcortical structures into 90 regions (pons and cerebellum excluded). Each region represented a node of the WM network. Briefly, the procedure included the following: (1) the structural image (i.e., T1-weighted image) of each participant was co-registered to their b0 image in the DTI native space by a linear transformation; (2) the co-registered image was non-linearly normalized to the T1 template of ICBM152 in the Montreal Neurological Institute (MNI) space, resulting in a nonlinear transformation; (3) the inverse transformation was applied to warp the AAL Template from the MNI space to each participant’s DTI native space.

Network Edge Definition The reconstruction of whole-brain WM tracts was implemented using the deterministic fiber assignment continuous tracking algorithm. If the turn angle of a fiber tract was greater than 45°or any voxel of a fiber’s fractional anisotropy became less than 0.2, the tracking procedure would be terminated. An edge was determined to exist if there were at least three tracts with terminal points between the two nodes. In this study, the weight of the edges was defined by the mean FA value (an index for evaluating WM fiber integrity) of the WM fibers between two nodes. Using these criteria, a 90 × 90 FA weighted metric was obtained for each subject.

PANDA was used to check the quality of the registrations, such as the registration of DTI to T1, the registration of T1 to MNI space, and the registration of DTI to MNI space. Track Vis (<http://www.trackvis.org/>) was used to check the quality of deterministic fiber tracking.

*Genotyping and Imputation*

SNPs with minor allele frequency (MAF) < 1%, call rate < 95% or Hardy–Weinberg equilibrium p < 10^-5^ were excluded from the analysis. We also excluded individuals with excessive missingness > 5%, gender mismatch, or an estimation of identity-by-descent > 0.90. After quality control, there were 8921608 imputed variants. Genotype imputation was performed by a commercial imputation engine named GenoImpute. We obtained a mean sample-level r^2^ of 0.736 estimated by 1% hold out SNPs on the array. This engine produces a continuous allele dosage and three genotype probability distribution which reflect the reality of genotype uncertainty.

Supplementary Table 1 Formulations and description of topological measurements applied in the study

| Network properties | Definitions | Measurement and meaning |
| --- | --- | --- |
| Nodal Degree, D_nodal_ |  | D_nodal_ is defined as the number of edges attached to the node.  D_nodal_ represents a node’s direct connection with other nodes in the network. |
| Nodal Efficiency, E_nodal_ | 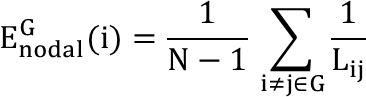 | E_nodal_ is defined as the average inverse of the shortest path length between node i and all the other nodes in the network.  Enodal represents the capacity of node i to communicate information with the other nodes in the network: A node with high E_nodal_ indicates great interconnectivity with other regions in the network |
| Global Network Efficiency, E_glob_ | 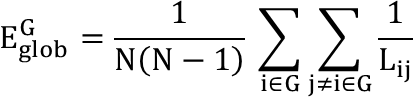 | E_glob_ is defined as the me an of the inverse of the shortest path length for all the nodes in the network.  E_global_ represents the global efficiency of parallel information transfer in the network. |
| Network Local Efficiency, E_loc_ | 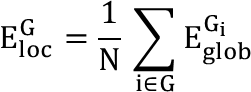 | E_loc_ is defined as the mean of local efficiencies for all the nodes in the network.  E_loc_ represents the ability to tolerate faults of the whole network. |

Supplementary Table 2 Demographic and clinical characteristics of SZ and GHR groups in SZ-PRS

|  | SZ | GHR | T/χ^2^ Value | *p* Value |
| --- | --- | --- | --- | --- |
| *n* | 26 | 48 |  |  |
| Demographic Characteristics |  |  |  |  |
| Age | 28.88(8.68) | 29.33(7.76) | -0.228 | 0.820 |
| Female, % | 69.2% | 43.0% | 3.717 | 0.054 |
| Clinical Characteristics |  |  |  |  |
| Duration of illness, months | 19.36(31.93) | — |  |  |
| First episode, yes% | 69.2% | — |  |  |
| Medication, yes% | 57.7% | — |  |  |
| HAMD | 7.00(6.06) | 2.59(3.68) | 3.268 | 0.003 |
| HAMA | 5.70(5.49) | 2.28(4.29) | 2.832 | 0.006 |
| BPRS | 31.92(11.90) | 18.96(2.14) | 5.401 | <.001 |

Data are presented as n (%) or mean (SD).

HAMD, Hamilton Depression Scale; HAMA, Hamilton Anxiety Scale; BPRS, Brief Psychiatric Rating Scale.

Supplementary Table 3 Differences in Topological Metrics among SZ, GHR and HC groups.

| Topological Metrics | Brain Region | HC | GHR | SZ | F Value | *P* Value | Bonferroni corrected *p* |
| --- | --- | --- | --- | --- | --- | --- | --- |
| **Asymmetry** |  |  |  |  |  |  |  |
| AI-E_nodal_ | Right superior frontal gyrus, orbital part | -9.22E-03(4.93E-02) | 1.29E-02(4.73E-02) | 2.72E-03(4.82E-02) | 7.192 | 0.001 | 0.039 |
| AI-E_glob_ |  | -4.64E-03(1.51E-02) | 1.97E-03(1.89E-02) | -2.79E-04(1.72E-02) | 4.245 | 0.015 | 0.075 |
| AI-E_loc_ |  | -3.72E-03(2.11E-02) | 3.02E-03(2.35E-02) | 2.02E-03(2.16E-02) | 5.783 | 0.003 | 0.017 |
| **Entire brain** |  |  |  |  |  |  |  |
| D_nodal_ | Left postcentral gyrus | 5.46E+00(1.15E+00) | 5.28E+00(1.17E+00) | 4.90E+00(1.12E+00) | 7.830 | <.001 | 0.042 |
| E_nodal_ | Right middle frontal gyrus | 2.09E-01(2.07E-02) | 2.08E-01(1.99E-02) | 2.00E-01(2.17E-02) | 7.672 | 0.001 | 0.049 |
|  | Right inferior frontal gyrus, opercular part | 2.12E-01(2.26E-02) | 2.10E-01(1.87E-02) | 2.02E-01(1.86E-02) | 8.392 | <.001 | 0.025 |
|  | Left superior occipital gyrus | 2.51E-01(2.34E-02) | 2.51E-01(2.61E-02) | 2.39E-01(2.80E-02) | 8.129 | <.001 | 0.032 |
|  | Left postcentral gyrus | 2.30E-01(2.02E-02) | 2.28E-01(2.01E-02) | 2.19E-01(2.04E-02) | 9.179 | <.001 | 0.012 |
|  | Right inferior parietal angular gyrus | 1.98E-01(1.57E-02) | 2.02E-01(1.76E-02) | 1.91E-01(1.92E-02) | 8.756 | <.001 | 0.017 |
|  | Left angular gyrus | 1.93E-01(1.67E-02) | 1.98E-01(1.87E-02) | 1.87E-01(1.78E-02) | 8.294 | <.001 | 0.027 |
| E_glob_ |  | 2.22E-01(1.18E-02) | 2.24E-01(1.46E-02) | 2.16E-01(1.59E-02) | 7.030 | 0.001 | 0.005 |
| E_loc_ |  | 3.03E-01(1.37E-02) | 3.08E-01(1.79E-02) | 2.98E-01(1.79E-02) | 6.755 | 0.001 | 0.007 |

Data are presented as mean (SD).
